# Supplementary material for: Enhancing Rural Healthcare Accessibility: A Model for Pharmacogenomics Adoption via an Outreach-Focused Integration Strategy
Source: J Pers Med. 2025 Mar 13;15(3):110. doi: 10.3390/jpm15030110 (PMC11943720; doi:10.3390/jpm15030110)
Supplement: Supplementary file 1 [file jpm-15-00110-s001.zip › jpm-3460168-supplementary.pdf]

Enhancing Rural Healthcare Accessibility: A Model for Pharmacogenomics Adoption via an Outreach-Focused Integration Strategy (Supplementary Materials)

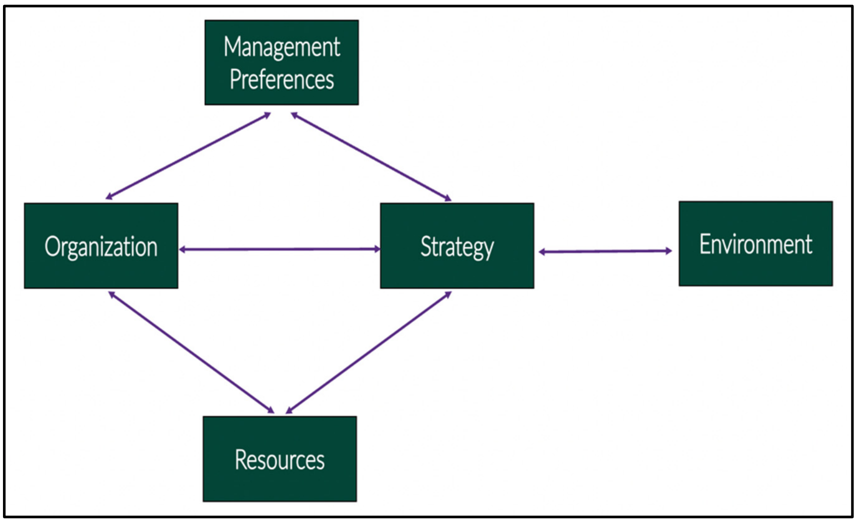

Figure S1. Diamond-E Framework Applied to Pharmacogenomic Implementation in Rural Healthcare

Note: This figure highlights the alignment between key operational elements, which informed the proposed action plan for rural healthcare accessibility. Adapted from (Crossan et al., 2015) [37].

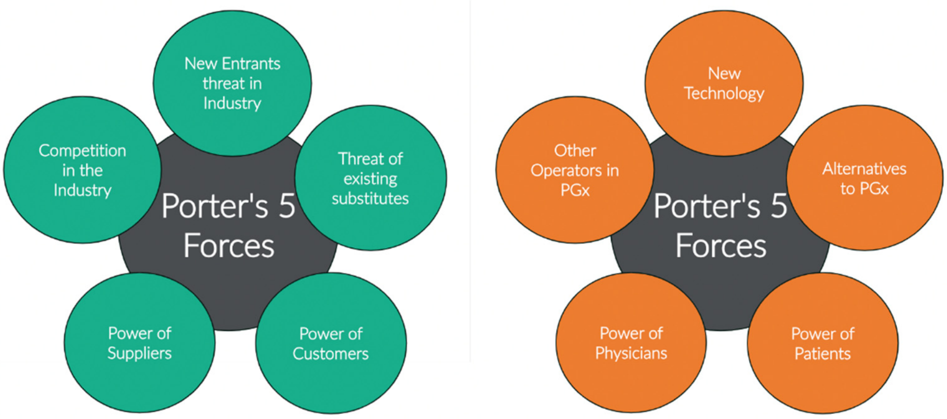

Figure S2. Porter's 5 Forces Analysis for Pharmacogenomic Accessibility

Note: This figure highlights each of the porter's 5 forces principles and how they were adapted to fit a healthcare setting. They were then used to understand where attention and resources are most needed for various implementations [13]. Adapted from (Porter, M. E., 2008)

Supplementary S1

Overview of Interviews Conducted

Table A1

Stakeholder Group	Number of Participants
Doctors/Residents	2
Pharmacists	3
Patient Partners	3

Supplementary S2

Semi-Structured Interview Guide for Healthcare Workers

Introduction and Context

- Briefly introduce yourself and express interest in understanding their perspective as a GP or Pharmacist.
- Explain the goal of gathering insights to enhance outreach strategies for pharmacogenomic services, especially looking at strategies that increase accessibility for rural patients.

<u>Open-Ended Questions:</u>

General Experience and Awareness

- "How familiar are you with pharmacogenomics and its applications in patient care?"
- "Can you describe any challenges you've faced when incorporating new medical advancements into your practice?"
- "What types of information or evidence would most persuade you to refer patients to pharmacogenomic services?"

Tailored Education and Training Needs

- "What educational materials or resources would be most helpful for you to confidently discuss pharmacogenomics with patients?"
- "Are there specific types of data or case studies that would make pharmacogenomics more tangible and relevant for your practice?"
- "How can outreach efforts (e.g., conferences, webinars, or targeted communication) be best aligned with your preferences and professional needs?"

Patient-Pharmacist/GP Collaboration

- "How do you currently communicate with patients about emerging technologies in medicine? Do you think similar methods would apply to pharmacogenomics?"
- "What additional support would you require to act as a liaison between patients and the pharmacogenomics service providers?"

Exploring Additional Improvements

- "Are there any areas of outreach or patient engagement that you feel are often overlooked in new healthcare initiatives?"

- "What would make pharmacogenomics adoption more streamlined and beneficial for both you and your patients?"
- "From your experience, what incentives or support systems work best to encourage the adoption of new healthcare technologies?"

Rural Application

- "How do you see mail-in testing for pharmacogenomics fitting into your workflow or practice?"
- "What is your perspective on using telemedicine to deliver pharmacogenomics consultations or follow-ups?"
- "Do you foresee any challenges with telemedicine services when dealing with pharmacogenomics, either for yourself or your patients?"
- "How could telemedicine be structured to ensure smooth communication and patient trust during virtual consultations?"
- "Are there specific factors that would increase your confidence in recommending mail-in kits to patients?"
- "What logistical or clinical concerns might arise for you when referring patients to use mail-in testing?"

Conclusion and Acknowledgment

- Thank them for their time and insights.
- Ask: "Is there anything else you believe is essential for us to know to improve our approach to introducing pharmacogenomics?"

Supplementary S3

Semi-Structured Interview Guide for Patient Parnters

Patient Experience Discussion

- Brief introduction and express interest in understanding their experiences as patients.
- Explain that you are seeking their input on specific recommendations to improve patient care, particularly focusing on mail-in testing and telemedicine services.

<u>**Open-Ended Questions:**</u>

- "Can you describe your experience as a patient, particularly focusing on any pharmacogenomic testing or telemedicine consultations you've undergone?"
- "What aspects of your patient experience did you find most positive or helpful?"
- "Were there any challenges or areas where you felt the care, or the process could be improved? Can you elaborate on those?"

Mail-in Testing

Briefly outline the proposed implementation of mail-in testing.

- "How far would you be willing to travel for this service?", If you had to travel xx distance, would mail-in be convenient."

- "How do you feel about the concept of mail-in testing for pharmacogenomic analysis? Do you see this as a beneficial addition to patient care?"
- "Are there any concerns or suggestions you have regarding the mail-in testing process, from your perspective as a patient?"
- "In what ways can we make the mail-in testing process more patient-friendly and accessible?"

Telemedicine Services

Describe how telemedicine services would be implemented, emphasizing the use of Zoom for Healthcare.

- "What are your thoughts on using telemedicine services like Zoom for healthcare consultations and follow-ups?"
- "Do you have any reservations or suggestions about telemedicine that could help tailor it better to patient needs?"
- "From your experience, how important is the ease of technology use in your healthcare journey, especially for services like telemedicine?"
- "I remember from the conference that keeping the patient feeling human is the most important thing. I want to make sure we can do that virtually. Are there any ways you could think of to do this?"

Exploring Additional Improvements

- "Beyond the mail-in testing and telemedicine services, are there other areas where you think patient care could be improved, especially for those living far from the clinic?"
- "What types of support or services do you think would be most beneficial for patients who don't live near the clinic?"
- "From your personal experience, what has been missing or could have made your healthcare journey smoother?"

Conclusion and Acknowledgment

- "Is there anything else you'd like to add or any other feedback you think would be important for us to consider in improving patient care?"

References

13. Porter, M. *The Five Competitive Forces That Shape Strategy*; Harvard Business School: Brighton, MA, USA, 2008.

37. Crossan, M.M.; Rouse, M.J.; Fry, J.N.; Rowe, W.G.; Maurer, C.C.; Killing, J.P. *Strategic Analysis and Action*; Pearson Education Canada: Toronto, ON, Canada, 2015.
